# Current perspectives in proteomic analysis of abiotic stress in Grapevines

**DOI:** 10.3389/fpls.2014.00686

**Published:** 2014-12-08

**Authors:** Iniga S. George, Paul A. Haynes

**Affiliations:** Department of Chemistry and Biomolecular Sciences, Macquarie UniversityNorth Ryde, NSW, Australia

**Keywords:** grape, proteomics, grapevine, abiotic stress

## Abstract

Grapes are an important crop plant which forms the basis of a globally important industry. Grape and wine production is particularly vulnerable to environmental and climatic fluctuations, which makes it essential for us to develop a greater understanding of the molecular level responses of grape plants to various abiotic stresses. The completion of the initial grape genome sequence in 2007 has led to a significant increase in research on grapes using proteomics approaches. In this article, we discuss some of the current research on abiotic stress in grapevines, in the context of abiotic stress research in other plant species. We also highlight some of the current limitations in grapevine proteomics and identify areas with promising scope for potential future research.

## Introduction

Grapes are a valuable fruit crop and wine production is a globally important industry with 265 million hectoliters of wine produced in 2011 (www.oiv.int) (2012). Grapevine production can be hampered by influential abiotic stresses like drought, climate fluctuations, and salinity. These factors pose a direct threat to viticulture practices. Global warming reports estimate an increase in temperature by 2–5°C by the end of the twenty-first century (Salinger, 2005), along with higher probability of stronger, more powerful, and more frequent climate fluctuations. The future beholds a warmer and more arid planet with sudden temperature fluctuations, caused by either natural or anthropogenic impacts. Global warming can lead to desertification, drought and intense soil salinity, all of which adversely affect grapevine quality and quantity. Studies have reported that abiotic stresses can impact wine grape production by decreasing yield and lowering quality of grapes produced (Jones et al., 2005; Cramer, 2010; Hannah et al., 2013). There is a forecast estimated decrease of up to 73% of surface land area suitable for viticulture in the main wine producing regions of the world by 2050 (Hannah et al., 2013). Although vineyards in some areas are adjusting to climate acclimation (Van Leeuwen et al., 2013), there is a need to develop environmentally sustainable crops without compromising on productivity and quality. This has driven much research into studies on plant responses to abiotic stresses. Proteomics using state of the art mass spectrometry is a powerful and promising tool to study molecular mechanisms and biological traits in plants. Grapevine responses to abiotic stresses like salt stress (Vincent et al., 2007; Jellouli et al., 2008), drought (Vincent et al., 2007; Grimplet et al., 2009b; Cramer et al., 2013), and temperature (Liu et al., 2014) have been effectively investigated. This short article will discuss the developments in grapevine proteomics, consider its current role in unraveling insights in molecular responses to abiotic stresses, and briefly discuss the current limitations of proteomic studies in grapevines.

## Developments in proteomics

Proteomic analysis techniques are constantly developing, with continuing improvements in sensitivity, resolution, accuracy, and speed of analysis. Advances in sample preparation techniques, mass spectrometry instrumentation and bioinformatics tools have paved the way for high throughput analysis. There have been great advancements in this field over the past two decades and these developments continue to expand, thus enhancing our understanding of molecular systems. In the past, sample preparation techniques using both in-gel digestion and in-solution digestion have been employed in proteomics studies on grape. Proteomic responses have been studied in tissues of grape berry (Sarry et al., 2004; Vincent et al., 2006; Grimplet et al., 2009b; Giribaldi et al., 2010; Martinez-Esteso et al., 2011b), leaf (Sauvage et al., 2007; Jellouli et al., 2010b; Delaunois et al., 2013; Nilo-Poyanco et al., 2013; Liu et al., 2014), stem (Jellouli et al., 2008), root (Castro et al., 2005; Jellouli et al., 2010b), shoot (Vincent et al., 2007; Cramer et al., 2013), and cell cultures (Martinez-Esteso et al., 2009, 2011c). Previously, two-dimensional gel electrophoresis techniques were mainly used (Vincent et al., 2007; Jellouli et al., 2008, 2010a; Grimplet et al., 2009b; Giribaldi and Giuffrida, 2010), but these are now being replaced by shotgun proteomics techniques including iTRAQ and TMT (Martinez-Esteso et al., 2011a; Liu et al., 2014), or label-free quantitation methods (Cramer et al., 2013), using ever more sophisticated mass spectrometers. Mass spectrometry instrumentation has evolved over the years from basic time-of-flight tandem mass spectrometers to multiplexed hybrid mass spectrometers. Instruments have become faster and more sensitive, with concomitant increases in resolution, thus generating far more data at higher accuracy. To keep up with these advancements, and the tremendous amount of data acquired, statistical software used to analyse mass spectrometry results, including from grapevine studies, has also been the subject of intense development. Statisticians, mathematicians and computer scientists have made efforts to create new and user friendly databases and algorithms to help understand molecular mechanisms. Consequently, the sequencing of the grape genome in 2007 (Jaillon et al., 2007; Velasco et al., 2007) represented a major breakthrough transition in grapevine proteomic research. The use of the *Vitis vinifera* genome sequence, containing approximately 30,000 genes, in database searches provided more reliable results than could be produced previously. Previous studies on grape have generally used the NCBI non-redundant database or EST contigs for identifying proteins (Marsh et al., 2010; Martinez-Esteso et al., 2011a) which works reasonably well but produces data which often represents an incomplete picture.

## Abiotic stress studies in different species

It is essential to produce sustainable plant varieties that adapt to climate variability, and to develop a broad spectrum of abiotic stress tolerant crops. Environmental factors influence dynamic changes in plants, often caused by either single or joint effects of numerous abiotic stress responsive pathways, that can be well characterized at the global level using high-throughput proteomic approaches. Proteomics has been successfully used to study abiotic stress responses in a wide range of plants like Arabidopsis (Rocco et al., 2013; Vialaret et al., 2014), rice (Neilson et al., 2013; Mirzaei et al., 2014), maize (Benesova et al., 2012), and poplar (Zhang et al., 2010), among many others, all of which have genomes that have been sequenced. This approach has also been employed for biomarker discovery in plant species with incomplete genome sequences, like peanut (Kottapalli et al., 2013), mango (Renuse et al., 2012), and even rare species like Pachycladon (Mirzaei et al., 2011), an Alpine species endemic to New Zealand. Among *Vitis vinifera* cultivars, proteomic studies prior to the sequencing of the grape genome relied on searching mass spectra against NCBI non-redundant protein databases or ESTs (Sarry et al., 2004; Vincent et al., 2006). The process of using mass spectrometers to identify proteins by cross species peptide identification is difficult, but it has become easier with the development of more high accuracy mass spectrometers.

Abiotic stress responses have been investigated in grapevine varieties of Chardonnay (Castro et al., 2005; Vincent et al., 2007), Tunisian Razegui (Jellouli et al., 2008), Cabernet Sauvignon (Vincent et al., 2007; Grimplet et al., 2009b; Cramer et al., 2013; Liu et al., 2014), and Pinot Noir (Negri et al., 2011). Grapevines have developed several adaptive approaches at the cellular and metabolic levels to mitigate, and recuperate from, the destructive effects of hostile environmental conditions. General responses include differential regulation of sugar metabolism, signaling, growth, protein synthesis, and hormone metabolism. As an example, we have observed changes in phenylpropanoid biosynthesis in Cabernet Sauvignon cells exposed to temperature stress (George et al., 2014). Osmotic stress response is the most common response to harsh environments (Cramer, 2010). Proteomics has aided in the study of differential expression of single proteins, global expression patterns, and association with regulatory pathways, and has also been used to substantiate and complement transcriptomic and metabolomic studies (Cramer et al., 2007; Zamboni et al., 2010). For example, a strong correlation was observed between transcriptomic and proteomic data in the investigation of biotic stress response to trunk diseases in green stems of Chardonnay (Spagnolo et al., 2012).

In order to better understand the metabolic changes involved in stress responses in both vegetative and reproductive parts of grapevines, and how dynamic the adaptative responses are in such situations, better experiments are needed. Ideally, if funding permitted, one would design experiments including sampling of all tissues—roots, shoots, leaves, and berries—at different developmental stages, including berry growth, veraison and ripening, and under various environmental conditions at different locations. Such an exhaustive study would be an invaluable resource for the grapevine research community, especially if it was expanded to include transcriptomic and metabolomic analysis in addition to the proteomic data sets.

## Current limitations of grapevine proteomics and future scope

With the genome sequences of various plants being completed and released regularly, it is becoming easier to examine the biological pathways that trigger plant protein responses. Studies in grape have increased exponentially since 2007, after the release of the grape genome sequence data. A vast amount of knowledge has been obtained from these studies on various defense mechanisms and biological pathways triggered by external factors, including both biotic, and abiotic stresses. Investigations have been performed on different varieties ranging from the widely recognized *Vitis vinifera* cultivars like Cabernet Sauvignon and Chardonnay (Vincent et al., 2007), to other species like *Vitis riparia* (Victor et al., 2010) and *Vitis rotundifolia* Michx Muscadine (Kambiranda et al., 2014). Despite these advances, detailed understanding of proteins and protein families which are essential for stress responses are still limited. The available grape genome sequence was based on analyses of only one *Vitis vinifera* variety, Pinot Noir PN40024. Most studies so far have used only the Pinot Noir genome sequence as the reference genome. It is well known that a significant amount of transcript and protein sequences are either species specific or cultivar specific and hence may not be well represented within the Pinot Noir genome. This may lead to incompleteness in protein identification when studying other grape varieties or species. Thus, there is a clear need for sequencing of more cultivars, such as the commercially important Cabernet Sauvignon, and more related species such as *V. rotundifolia* and *V. riparia*. There are now hundreds of genome sequences available for different ecotypes of Arabidopsis (Weigel and Mott, 2009), and with the continued rapid developments in gene sequencing technologies we would hope that in the near future we will also see the publication of complete genome sequence information data for many different varieties of grapevine.

A critical challenge in grapevine proteomics is to infer biological meaning from the huge amount of mass spectrometry data acquired. The general procedure for the study of plant-environment interactions includes protein identification, protein characterization (including function annotation), construction of identified proteins into a biological network, characterization of differential protein expression under stress conditions, and assimilation of all the above into a linking framework. The initial step for this type of workflow is to identify and annotate proteins, and integrate them into the biological context.

In order to illustrate some of the current difficulties in this process, we surveyed grapevine protein sequences in NCBI and Uniprot, using the simple keyword “*Vitis vinifera*.” We found 161926 sequences in NCBI and 65548 sequences in Uniprot, which is indicative of a high level of redundancy and repetition, particularly in the NCBI database. We examined the entries in the Uniprot database in more detail. Table 1 shows the number of protein entries for different grapevine species found in the Uniprot database, along with an indication of how many of these are still uncharacterized. Most protein entries in the database are unreviewed, which means that no additional supporting information has been presented for them. Moreover, 78% of the protein entries in the *Vitis vinifera* database are listed as “putative uncharacterized proteins.” Hence, proteomic study is severely limited by the lack of better quality annotations.

**Table 1:** **Number of protein entries in the UniprotKB database for different grapevine species**.

**Species**	**UniprotKB entries**	**No. of reviewed proteins**	**No. of unreviewed proteins**	**No. of putative uncharacterized proteins**
*Vitis vinifera*	65548	206	65342	50775
*Vitis rupestris*	536	3	533	1
*Vitis labrusca*	195	1	194	5
*Vitis riparia*	178	2	176	2
*Vitis vulpina*	167	2	165	2
*Vitis amurensis*	155	0	155	0
*Vitis rotundifolia*	111	2	109	0
*Vitis aestivalis*	66	3	63	0
*Vitis coignetiae*	7	0	7	0

In previous studies, since the roles of many individual proteins were not well defined, their biological functions were inferred from homologous proteins from other species. This task is tedious and time consuming, and produces less than complete protein identification data. Although grapevine does not have a well-established database like PPDB (Sun et al., 2009) (which is dedicated exclusively for *Ar*a*bidopsis thaliana* and *Zea mays* research), there is a basic database developed uniquely for grapevine molecular network study called VitisNet (Grimplet et al., 2009a). VitisNet was developed from the combination of *Vitis vinifera* (cv. Pinot Noir PN40024) genome sequencing project data, and ESTs from the *Vitis* genus, and is very useful for annotating grapevine proteins (Grimplet et al., 2012). PlantPReS (not yet published) (http://proteome.ir/PlantStress.aspx) is a freely available plant stress protein database which integrates different plant proteomic responses to stress studies. It currently has 83 plant species and is inclusive of *Vitis vinifera*. This database is still under construction, but the data that have been incorporated so far have proved useful in annotating grapevine proteins identified in proteomics experiments.

There is a pressing need to enable the integration of large datasets, streamline biological functional processing, and improve the understanding of dynamic processes in systems biology experiments in grapevines. At the moment, software packages available for this purpose are mainly designed to work with mammalian systems. It is to be hoped that in the future more software is available that is specifically designed to function with plant protein and genome sequences, including grapevines.

## Conclusion

Proteomics is a powerful tool for molecular level discovery of biological networks in grapevine. Plant species with completely sequenced genomes, smaller genome sizes and well annotated libraries are easier to study and understand; grapevines remain a challenge. Recent advancements in mass spectrometry and proteomic techniques, coupled with the availability of complete genome sequences and improvements in bioinformatics tools, are continually strengthening this field of study. Research in this area, however, needs to be further accelerated by sequencing more grapevine varieties and different cultivars. Protein sequences in database repositories need much more functional annotation, which will help obtain better results and a more comprehensive understanding of biological responses. Proteomics has an important role to play in the future in helping to understand at the molecular level how grapevines respond to the many challenges they face.

### Conflict of interest statement

The authors declare that the research was conducted in the absence of any commercial or financial relationships that could be construed as a potential conflict of interest.
